# Prevalence of Anxiety among Hungarian Subjects with Parkinson's Disease

**DOI:** 10.1155/2017/1470149

**Published:** 2017-09-26

**Authors:** Márton Kovács, Attila Makkos, Rita Weintraut, Kázmér Karádi, József Janszky, Norbert Kovács

**Affiliations:** ^1^Department of Neurology, University of Pécs, Pécs, Hungary; ^2^MTA-PTE Clinical Neuroimaging MR Research Group, Pécs, Hungary

## Abstract

Although anxiety is one of the most frequent symptoms of Parkinson's disease (PD), only a few clinical tools can efficiently and reliably detect its presence. The aim of the present study was to validate the Hungarian patient-rated version of Parkinson Anxiety Scale (PAS). A total of 190 PD patients were enrolled into the clinimetric validation phase of the study and another 590 participated in the cross-sectional screening phase. The presence of anxiety disorder was diagnosed based on the Diagnostic and Statistical Manual of Mental Disorders criteria. The cutoff value for PAS which best discriminated the presence of anxiety from the absence was 12.5 points (sensitivity of 88.6%, specificity of 79.9). The area under the curve was 0.847 whereas the ROC analysis yielded the statistical significance level (*p* < 0.001). The optimal threshold values for mild (Hoehn and Yahr Stage, HYS 1 and 2), moderate (HYS 3), and severe (HYS 4 and 5) disease stages were 10.5, 12.5, and 13.5 points, respectively. Based on the general threshold anxiety occurred in 35.8% of the patients (persistent anxiety: 29.2%, episodic anxiety: 20.7%, and avoidant anxiety disorder: 16.8%). We demonstrate that the PAS is a valid, highly reliable, and sensitive tool for assessing anxiety.

## 1. Introduction

Among the nonmotor symptoms of Parkinson's disease (PD), the affective problems have utmost importance. According to epidemiological studies, the frequency of anxiety disorders is higher than that of depressive problems being between 25–49% [1, 2]. The spectrum of anxiety is extremely wide including panic attacks, agoraphobia without panic disorder, social phobia, and generalized anxiety disorder. Besides the general problems of anxiety, PD patients frequently experience disease-specific symptomatology including distress, worry, fear, agitation, embarrassment, and social withdrawal due to motor symptoms and complications of antiparkinsonian medication [3, 4]. Besides producing immense suffering, anxiety has a serious impact on the daily living and health-related quality of life (HRQoL) [5].

In the clinical setup, reliable and highly sensitive tools are required to identify anxiety problems and monitor the therapeutic response. Commonly applied instruments, such as Hamilton Anxiety Rating Scale (HAM-A), the Beck Anxiety Inventory, and the Hospital Anxiety and Depression Scale, have unsatisfactory discriminating abilities [6]. With the help of The Michael J. Fox Foundation, the Parkinson Anxiety Scale (PAS) was recently developed and validated to overcome the limitation of the abovementioned anxiety scales and to provide a license-free clinical and research tool for the PD population [7].

The PAS is a 12-item tool which can be either rated by a trained professional (observer version) or by the patients themselves (patient-rated version). The PAS make up three different subscales describing the persistent anxiety (5 items), episodic anxiety (4 items), and avoidance behavior (3 items) [7]. Each item can be scored on a 5-point Likert scale, with “0” meaning “not or never” and “4” meaning “severe or almost always” implying a maximum of total score of 48 points. According to its developers, the assessment of the self-rated version can be completed in less than 2 minutes, whereas the observer-rated version may take up to 5 minutes. Based on a multinational, multicenter, and cross-sectional validation study enrolling 360 PD patients, the PAS had better clinimetric properties than any other existing scale making it a brief and nevertheless valid and highly reliable tool.

The PAS scale was originally developed in four languages simultaneously (English, Spanish, French, and Dutch) and subsequently translated into Italian [8]. Since the prevalence and perceived difficulties associated with anxiety differ among cultures, the cross-cultural validation of the PAS is clearly needed. Because Hungarian is a Finno-Ugric language originating from the Uralic language family vastly different from the Anglo-Saxon and Latin languages, we aimed to validate the Hungarian version of the PAS. Subsequently, we utilized this Hungarian version of PAS scale for determining the prevalence and severity of anxiety in a large pool of Hungarian subjects with PD in a cross-sectional study.

## 2. Materials and Methods

### 2.1. Patients

While in the validation part of the study, 190 consecutive patients fulfilling the clinical criteria for PD were enrolled, another 590 PD subjects participated in the subsequent part assessing the prevalence of anxiety. Each subject gave written informed consent in accordance with the ethical approval of the Regional and Institutional Ethical Committee of the University of Pécs (5624/2015), and subsequently they were examined by a neurologist specializing in movement disorders. Besides recording demographic data (age, sex, and level of education), disease-specific data were noted (age at onset; disease duration; the presence of motor complications; duration of fluctuation in years; type of PD, being either tremor dominant, akinetic rigid, or mixed type; and antiparkinsonian medication). Patients were evaluated in ON state while receiving their usual antiparkinsonian and other medications, and subsequently levodopa equivalent dosage (LED) calculations were performed [9].

### 2.2. Validation of the Hungarian Parkinson Anxiety Scale

Two native Hungarian speakers fluent in English translated independently the PAS into Hungarian, and an English-speaking psychologist back-translated the PAS into English. Subsequently, the original and the back-translated versions were compared and any discrepancies were fixed to achieve the first Hungarian patient-rated and observer-rated PAS. Cognitive debriefing was applied on 25 patients before field testing.

The self-rated version of PAS was administered to 190 consecutive PD patients. Exclusion criteria for participation were the following:

1. Clinical diagnosis of PD in accordance with the UK Brain Bank criteria could not be met [10]
2. Presence of major neurocognitive disorder in accordance with the Diagnostic and Statistical Manual of Mental Disorders (Fifth edition, DSM-5) criteria [11]
3. The presence of any neurological or psychiatric condition possibly interfering the study

### 2.3. Neurological and Neuropsychological Assessments

The severity of PD-related symptoms was globally assessed by the Hungarian-validated version of the MDS-UPDRS [12, 13]. The recently published MDS-UPDRS is a validated scale to assess nonmotor (nM-EDL, Part I) and motor experiences of daily living (M-EDL, Part II), motor examination (ME, Part III), and motor complications (MC, Part IV) [13]. As a part of the MDS-UPDRS, the Hoehn and Yahr Scale (HYS) was also taken to detect the overall severity of PD. Disease severity was categorized as mild (HYS 1 and 2), moderate (HYS 3), and severe (HYS 4 and 5) [14, 15].

The nM-EDL part of the MDS-UPDRS has items evaluating the presence and severity of 13 NMS including depression and anxiety [13]. These items are intended to serve as screening tools for the presence of these nonmotor symptoms [16]. To assess nonmotor symptoms globally, the Nonmotor Symptoms Scale (NMSS) [17, 18] was also included. This scale is obtained by trained professionals and capable of simultaneously capturing the severity and frequency of 30 nonmotor symptoms typical for PD. Severity of depression was assessed by the Hungarian-validated versions of the Montgomery Depression Scale (MADRS) [19] and the Beck Depression Inventory; whereas, the severity of anxiety was measured by the Hamilton Anxiety Scale (HAM-A) [20] and the PAS, and apathy was rated by the Lille Apathy Rating Scale (LARS) [21].

Patients were screened for the presence of mild and major neurocognitive disorders by validated neurocognitive tests [22] (Montreal Cognitive Assessment, cutoff scores <23.5 and <20.5, respectively, and Mattis Dementia Rating Scale, cutoff scores <139.5 and <132.5 points, resp.) [23, 24]. Health-related quality of life (HRQoL) was measured by the Hungarian-validated version of the disease-specific PDQ-39 Summary Index (PDQ-39 SI) [25, 26]. Subsequently, the presence of anxiety in accordance with the DSM-5 criteria [11] was assessed by a trained neuropsychiatrist in the validation phase.

### 2.4. Statistical Analysis

For variables following the normal distribution (e.g., age, disease duration), means ± standard deviations (SD) were calculated. Data quality was defined as the proportion of computable data. The criterion for an acceptable amount of missing data is <10% [27]. For acceptability, the floor and ceiling effect should be kept <15% [28], and the skewness should range between −1 and +1 [29].

Before the structure of the scale was explored by a factor analysis, the value of Kaiser-Meyer-Olkin measure of sampling accuracy (KMO) was calculated. A KMO >0.60 is a minimum requirement; whereas, KMOs >0.90 are considered as excellent for factor analysis. We accepted only those factors having an eigenvalue >1 and a Scree test for factor analysis.

In the clinimetrics, reliability is the overall consistency of a measure. A measure is said to have a high reliability if it produces similar results under consistent conditions [30]. In our study, the internal consistency was evaluated by four different approaches [31]:

- Cronbach's *α* should be >0.70 [32].
- Corrected item-total correlation should be >0.30 for each item.
- Item homogeneity coefficient should be >0.30.
- Test-retest properties (Intraclass Correlation Coefficient, ICC should be >0.6) [33]. The retest properties of the PAS were analyzed on a subset of patients (*n* = 89) one day after the initial examination.

The validity of an assessment is the degree to which it measures what it is supposed to measure. Therefore, it corresponds to how a measurement is well founded and accurately describes the real world [30]. In our study, the construct validity was evaluated by three different methods:

- Convergent validity: convergent validity refers to the degree to which a measure is correlated with other measures that it is theoretically predicted to correlate [30]. The total score and the subscores of PAS were compared to the “Anxiety” item of MDS-UPDRS and HAM-A. For correlation, Spearman's rank correlation coefficients were calculated. The values of correlation coefficients (*r*_S_) can indicate weak (0–0.299), moderate (0.300–0.599), and high (0.600–1.000) association [34].
- Internal validity: the correlation between the domains (subscales) should not be too low (*r*_S_< 0.300) or too high (*r*_S_> 0.700) either [35].
- Divergent validity: divergent validity tests whether concepts or measurements that are supposed to be unrelated are, in fact, unrelated [30]. We tested the discriminative validity of the PAS against apathy (Lille Apathy Rating Scale), depressive syndromes (BDI and MADRS), and cognitive functioning (MoCA) by calculating Spearman rank correlation coefficients [7].

The precision of the PAS was estimated by the standard error of measurement (SEM), where the value of SEM should be less than the half of the standard deviation.

In order to establish a cutoff value for the total score of the PAS, which can reliably differentiate PD patients with and without anxiety, we applied receiver operating curve (ROC) analysis. Patients were categorized by the DSM-5 criteria for anxiety disorders. This categorization served as the state variable and the PAS total score as the test variable. The best cutoff value was estimated as the point on the ROC curve closest to the point of (0.1). It was calculated as the minimum value of the square root of (1 − sensitivity)^2^ + (1 − specificity)^2^. Besides, the area under the curve, specificity, sensitivity, and positive and negative likelihood ratios were calculated for the best cutoff value. After establishing the optimal threshold for the whole population, separate cutoff limits were calculated for the different disease severity categories.

All statistical analyses were carried out using IBM SPSS software package (version 24.0.0.1, IBM Inc., Chicago, USA). Statistical significance level was set to 5%. Because the SPSS Suite did not have built-in functions for calculating positive and negative likelihood ratios, we utilized the syntax available on the IBM website (http://www-01.ibm.com/support/docview.wss?uid=swg21483380, assessed on Jan 15, 2013).

### 2.5. Determining the Prevalence of Anxiety among Hungarian PD Patients

Utilizing the newly determined cutoff score for the presence of anxiety, a large pool of PD patients were screened (*n* = 590). The presence and severity of anxiety was assessed by the patient-rated version of PAS.

## 3. Results

### 3.1. Validation of the Hungarian Parkinson Anxiety Scale

The subject population consisted of 190 PD patients without major neurocognitive disorder, and their clinical characteristics are demonstrated in Table 1. As for the quality of data, there was no missing data. The skewness of the scale was within the limit (0.828). Only a few patients had a total score of 0 on PAS (4.6%) and no one achieved a maximum score; therefore, the floor and ceiling effects were acceptable.

The KMO value was sufficiently high (0.909) to enable a factor analysis. The Scree test supported a three-factor solution explaining 67.6% of the variance. Using the Principal Component Analysis extraction method with Promax rotation, we identified the same factor structure as it was originally described.

The value of Cronbach's *α* for the total score and the persistent, episodic, and avoidance domains was acceptable (0.935, 0.897, 0.828, and 0.724, resp.). All the items reached the 0.30 threshold value for the item-total correlation. Item homogeneity index values were acceptable for all subdomains and the total score of PAS. The total score of PAS demonstrated high Spearman's rank correlation coefficient with both HAM-A and MDS-UPDRS “Anxiety” item (0.618 and 0.602). The internal validity for the subdomains of PAS was acceptable (*r*_S_ values in the range of 0.300–0.700). The test-retest validity was also acceptable (ICC = 0.824). As far as the discriminative properties were considered, the total score of PAS had poor correlation with LARS (rho = 0.226, *p* < 0.05), MoCA (rho = −0.185, *p* < 0.05), and moderate correlation with depression (MADRS rho = 0.536, *p* < 0.05, and BDI rho = 0.586, *p* < 0.05, overall population). In patients without anxiety, the rho values were 0.219 and 0.317 for MADRS and BDI; whereas, in the presence of anxiety, these values increased to 0.504 and 0.584, respectively. These discriminant values were similar to those of the original PAS validation study [7] and the Italian language validation study [8]. The precision was acceptable for both the domains and the total score of PAS.

Based on the DSM-5 criteria, 78 patients (41.1%) had an anxiety disorder. Generalized anxiety was found in 52 (27.3%), agoraphobia and social phobia in 48 patients (25.3%), and panic disorder in 31 patients (16.3%).

The cutoff value for PAS which best discriminated the presence of anxiety from the absence was 12.5 points. Therefore a PAS score ≥ 13 points may suggest the presence of anxiety (sensitivity of 88.6%, specificity of 79.9%, positive likelihood ratio: 2.64, and negative likelihood ratio: 0.17). The area under the curve (AUC) was 0.847 whereas the ROC analysis yielded the statistical significance level (*p* < 0.001). The optimal threshold values for mild, moderate, and severe disease stages were slightly different (10.5, 12.5, and 13.5 points, resp.). Subsequently, we also calculated the most optimal threshold values for each subscale (Table 2).

### 3.2. Prevalence of Anxiety among Hungarian PD Patients

In most cases, it took approximately 2–5 minutes to administer the scale if the patients completed by themselves. Based on the general threshold (12.5 points), anxiety occurred in 211 patients (35.8%). While persistent anxiety was found in 172 (29.2%), only 122 patients (20.7%) had episodic anxiety and another 99 patients (16.8%) had an avoidant anxiety disorder. Demographic and disease-specific data of the PD patients with and without anxiety are demonstrated in Table 3. Patients with anxiety had more severe PD-related symptoms (higher scores on all domains of MDS-UPDRS), depressive symptoms (BDI and MADRS), worse neurocognitive performance (MDRS and MoCA), and worse HRQoL (measured by PDQ-39).

## 4. Discussion

The aim of the present study was to validate the Hungarian patient-reported version of the PAS by assessing its fundamental clinimetric properties and subsequently determining the prevalence and severity of anxiety among Hungarian PD patients. After a standardized translation and back-translation of the scale, we initiated a hospital-based validation study on a large diversity of patients having disease severity from minimal to severe. Our results demonstrated excellent data quality, high reliability and validity, and good precision. These findings are consistent with the results of the original [7] and the Italian [8] validation studies.

The original validation study revealed the cutoff score of 13.5 points for the most optimal discrimination between the PD patients having and not having an anxiety disorder for both the patient-reported and observed-rated versions. However, a considerably smaller threshold (8.5 points) was calculated in the Italian validation study for the observer-rated version. This inconsistency was presumably contributed to some clinical and methodological factors. While the Italian sample included only patients with early disease stages (HYS 1 and 2), the original validation study not only had higher sample size (362 versus 101) but their subjects also had longer disease duration (10.4 versus 8.3 years), wider disease spectrum (HYS 1–4), and more pronounced Parkinsonian symptoms (UPDRS score: 24.7 versus 13.3 points). Moreover, the prevalence rate of anxiety disorders diagnosed by the DSM criteria used as “gold standard” was also dissimilar between the two aforementioned validation studies (27% versus 38.6%).

Our calculated threshold value (12.5) is much closer to that of the original validation study (13.5 points). Acknowledging the conclusions of the Italian validation study by hypothesizing the role of disease severity in the differences of anxiety threshold value, we included PD patients with a high diversity of disease stages (HYS 1–5) and calculated distinct threshold values for the different disease severity stages.

Based on the obtained cutoff threshold, we identified the prevalence of anxiety among Hungarian PD patients of which 35.8% is in the range of the internationally published range [1, 2]. Similar to previous findings, we also demonstrated that the presence of anxiety was associated with worse motor performance, cognitive performance, and more severely impaired health-related quality of life [36, 37].

## 5. Conclusions

The Hungarian patient-rated version of the Parkinson Anxiety Scale is a valid, highly reliable, and sensitive tool for assessing the presence and severity of the anxiety symptoms. Although our uniform threshold value may efficiently identify patients with anxiety, different threshold values may be utilized for different disease stages.

## Supplementary Material

The validated Hungarian Parkinson Anxiety Scale.

## Figures and Tables

**Table 1:** Demographic and disease-specific data of the study population (*n* = 190) participating in the validation phase.

	Mean or count	Standard deviation or percentage
Age (years)	65.8	9.8
Sex		
Male	110	57.9%
Female	80	42.1%
Education (years)	12.5	3.2
Disease duration (years)	7.2	6.4
Disease duration (years)	7.2	6.4
Type of disease		
Tremor dominant	61	32.1%
Akinetic rigid	79	41.6%
Mixed	50	26.3%
Hoehn and Yahr Stage		
Mild (1 & 2)	109	57.3%
Moderate (3)	43	22.7%
Severe (4 & 5)	38	20.0%
Levodopa dosage (in LED mg)	472.8	510.1
Dopamine agonist usage (in LED mg)	165.8	219.3
Antiparkinson medication (in LED mg)	677.5	600.6
MDS-UPDRS nM-EDL	13.1	7.5
MDS-UPDRS M-EDL	13.9	9.1
MDS-UPDRS ME	35.0	15.9
MDS-UPDRS MC	4.4	3.4
Nonmotor Symptoms Scale	53.9	38.8
Montreal Cognitive Assessment	22.7	4.4
Beck Depression Inventory	11.7	8.8
Lille Apathy Rating Scale	−22.5	9.5
Hamilton Anxiety Scale	13.1	6.7
Parkinson Anxiety Scale (Part A)	7.0	4.6
Parkinson Anxiety Scale (Part B)	2.4	2.9
Parkinson Anxiety Scale (Part C)	1.9	2.3
Parkinson Anxiety Scale (total score)	11.3	8.4

LED = levodopa equivalent dosage; MDS-UPDRS = Movement Disorders Society-sponsored version of Unified Parkinson's Disease Rating Scale; MDS-UPDRS M-EDL = motor experiences of daily living (Part II of MDS-UPDRS); MDS-UPDRS nM-EDL = nonmotor experiences of daily living (Part I of MDS-UPDRS); SD = standard deviation.

**Table 2:** Calculation of the optimal cutoff levels for detecting anxiety based on receiver operating curve analysis.

Scale	Clinical correspondence	Cutoff	Sensitivity	Specificity	AUC	*p* value
Total score of PAS	Any anxiety disorders	12.5	88.6%	79.9%	0.847	*p* < 0.001
Persistent anxiety subscale	Generalized anxiety disorder	9.5	89.3%	81.2%	0.875	*p* < 0.001
Episodic anxiety subscale	Panic disorder	4.5	92.1%	81.5%	0.921	*p* < 0.001
Avoidant anxiety subscale	Avoidant anxiety disorders	3.5	78.4%	82.4%	0.835	*p* < 0.001

Anxiety disorders characterized by avoidance are agoraphobia and social phobia (here taken together as avoidant anxiety disorders). ROC = receiver operating characteristics; AUC = area under the curve; PAS = Parkinson Anxiety Scale.

**Table 3:** Comparison of the clinical profile of the Parkinson's disease patients (*n* = 590) with and without anxiety based on the established Parkinson Anxiety Scale threshold value.

	No anxiety (*n* = 379)		Presence of anxiety (*n* = 211)		Statistics
	Mean or count	SD or percentage	Mean or count	SD or percentage	
Age (years)	65.8	9.7	65.9	9.9	NS
Disease duration (years)	7.2	6.1	7.2	7.1	NS
Sex					
Male	248	65.4%	92	43.6%	*p* < 0.001
Female	131	34.6%	119	56.4%	
Levodopa dosage (in LED mg)	446.7	517.6	517.3	495.0	NS
Dopamine agonist usage (in LED mg)	175.2	212.6	149.7	229.8	NS
Antiparkinson medication (in LED mg)	659.5	600.4	708.0	601.2	NS
Parkinson Anxiety Scale (total score)	6.1	3.7	20.2	6.6	*p* < 0.001
Parkinson Anxiety Scale (Part A)	4.3	2.8	11.5	3.2	*p* < 0.001
Parkinson Anxiety Scale (Part B)	0.9	1.2	5.0	3.1	*p* < 0.001
Parkinson Anxiety Scale (Part C)	0.8	1.2	3.7	2.7	*p* < 0.001
Hamilton Anxiety Scale	10.6	5.7	17.3	6.2	*p* < 0.001
Beck Depression Inventory	7.7	5.8	18.5	8.8	*p* < 0.001
Montgomery-Asberg Depression Rating Scale	8.9	6.0	16.2	7.2	*p* < 0.001
Nonmotor Symptoms Scale	40.5	30.0	76.5	41.5	*p* < 0.001
Montreal Cognitive Assessment	23.2	4.1	21.8	4.7	*p* < 0.05
Mattis Dementia Rating Scale	135.8	8.0	133.7	10.4	*p* < 0.05
Lille Apathy Rating Scale	−24.1	8.3	−19.8	10.9	*p* < 0.001
Parkinson's Disease Questionnaire	16.7	12.2	32.6	16.8	*p* < 0.001
MDS-UPDRS nM-EDL	10.4	6.2	17.8	7.2	*p* < 0.001
MDS-UPDRS M-EDL	11.8	8.1	17.5	9.6	*p* < 0.001
MDS-UPDRS ME	33.2	15.4	38.0	16.3	*p* < 0.001
MDS-UPDRS MC	3.8	3.2	5.3	3.4	*p* < 0.001
MDS-UPDRS total score	59.2	26.8	78.4	29.6	*p* < 0.001
Antidepressant usage	72	19.0%	178	84.3%	*p* < 0.001
Anxiolytics usage	87	22.9%	162	76.7%	*p* < 0.001

LED = levodopa equivalent dosage; MDS-UPDRS = Movement Disorders Society-sponsored version of Unified Parkinson's Disease Rating Scale; MDS-UPDRS M-EDL = motor experiences of daily living (Part II of MDS-UPDRS); MDS-UPDRS nM-EDL = nonmotor experiences of daily living (Part I of MDS-UPDRS); SD = standard deviation.

## Abbreviations

AUC: Area under the curve
BDI: Beck Depression Inventory
DSM-5: Diagnostic and Statistical Manual of Mental Disorders, Fifth edition
HAM-A: Hamilton Anxiety Scale
HYS: Hoehn and Yahr Stage
ICC: Intraclass Correlation Coefficient
LARS: Lille Apathy Rating Scale
LED: Levodopa equivalent dosage
MADRS: Montgomery-Asberg Depression Rating Scale
MDS-UPDRS: Movement Disorders Society-sponsored version of Unified Parkinson's Disease Rating Scale
MDS-UPDRS MC: Motor complications (Part IV of MDS-UPDRS)
MDS-UPDRS ME: Motor examination (Part III of MDS-UPDRS)
MDS-UPDRS M-EDL: Motor experiences of daily living (Part II of MDS-UPDRS)
MDS-UPDRS nM-EDL: Nonmotor experiences of daily living (Part I of MDS-UPDRS)
MoCA: Montreal Cognitive Assessment
PD: Parkinson's disease
PAS: Parkinson Anxiety Scale
ROC: Receiver operation curve analysis
SD: Standard deviation.
