# Supplementary material for: Prevalence of Anxiety among Hungarian Subjects with Parkinson's Disease
Source: Behav Neurol. 2017 Sep 26;2017:1470149. doi: 10.1155/2017/1470149 (PMC5634583; doi:10.1155/2017/1470149)
Supplement: Supplementary file 1 — The validated Hungarian Parkinson Anxiety Scale. [file 1470149.f1.pdf]

PAS skála

Név:...

Születés:...................................

Dátum:.....................................

A. Tartós szorongás

Kérjük, hogy minden kérdésnél kizárólag egy választ jelöljön meg!

Az elmúlt négy hét során milyen mértékben észlelte az alábbi tüneteket?

A.1. Szorongás vagy idegesség érzése

- ◻ 0. Egyáltalán nem vagy sohasem
- ◻ 1. Igen csekély fokban vagy ritkán
- ◻ 2. Enyhe fokban vagy néha
- ◻ 3. Közepes fokban vagy gyakran
- ◻ 4. Súlyos fokban vagy (szinte) mindig

A.2. Feszültség vagy stressz érzése

- ◻ 0. Egyáltalán nem vagy sohasem
- ◻ 1. Igen csekély fokban vagy ritkán
- ◻ 2. Enyhe fokban vagy néha
- ◻ 3. Közepes fokban vagy gyakran
- ◻ 4. Súlyos fokban vagy (szinte) mindig

A.3. Nem tud ellazulni

- ◻ 0. Egyáltalán nem vagy sohasem
- ◻ 1. Igen csekély fokban vagy ritkán
- ◻ 2. Enyhe fokban vagy néha
- ◻ 3. Közepes fokban vagy gyakran
- ◻ 4. Súlyos fokban vagy (szinte) mindig

A.4. Kifejezett aggódás a hétköznapi ügyek miatt

- ◻ 0. Egyáltalán nem vagy sohasem
- ◻ 1. Igen csekély fokban vagy ritkán
- ◻ 2. Enyhe fokban vagy néha
- ◻ 3. Közepes fokban vagy gyakran
- ◻ 4. Súlyos fokban vagy (szinte) mindig

A.5. Valami rossz, vagy akár a legrosszabb, dolog megtörténésétől való félelem

- ◻ 0. Egyáltalán nem vagy sohasem
- ◻ 1. Igen csekély fokban vagy ritkán
- ◻ 2. Enyhe fokban vagy néha
- ◻ 3. Közepes fokban vagy gyakran
- ◻ 4. Súlyos fokban vagy (szinte) mindig

B. Időszakosan jelentkező szorongás

Kérjük, hogy minden kérdésnél kizárólag egy választ jelöljön meg!

Az elmúlt négy hét során észlelt –e az alábbi tünetekkel járó rosszullétet?

B.1. Pánik vagy heves félelem

- ◻ 0. Sosem
- ◻ 1. Ritkán
- ◻ 2. Néha
- ◻ 3. Gyakran
- ◻ 4. Szinte mindig

B.2. Légzési nehezítettség

- □ 0. Sosem
- □ 1. Ritkán
- □ 2. Néha
- □ 3. Gyakran
- □ 4. Szinte mindig

B.3. Szívdobogásérzés vagy szapora szívverés (mely nem kapcsolatos fizikális megterheléssel vagy cselekvéssel)

- □ 0. Sosem
- □ 1. Ritkán
- □ 2. Néha
- □ 3. Gyakran
- □ 4. Szinte mindig

B.4. Önuralom elvesztésétől való félelem

- □ 0. Sosem
- □ 1. Ritkán
- □ 2. Néha
- □ 3. Gyakran
- □ 4. Szinte mindig

<u>C. Elkerülő magatartás</u>

Kérjük, hogy minden kérdésnél kizárólag egy választ jelöljön meg!

Az elmúlt négy hétben milyen mértékben érzett félelmet az alábbi helyzetekben vagy kerülte el az alábbi helyzeteket?

C.1. Társasági helyezetek (ahol mások is megfigyelhetik és kritizálhatják, úgymint közönség előtt beszélgetés vagy ismeretlenekkel történő társalgás)

- □ 0. Sosem
- □ 1. Ritkán
- □ 2. Néha
- □ 3. Gyakran
- □ 4. Szinte mindig

C.2. Közösségi helyezetek (olyan helyzetek, ahonnan nehéz vagy pedig zavaró lenne elmenekülni, úgymint sorban állás alatt, tömegben, hidakon vagy tömegközlekedés során)

- □ 0. Sosem
- □ 1. Ritkán
- □ 2. Néha
- □ 3. Gyakran
- □ 4. Szinte mindig

C.3. Speciális helyzetek vagy tárgyak (például repülés során, magasban tartozkodás, pókok vagy egyéb állatok látványa, tűk vagy a vér látványa)

- □ 0. Sosem
- □ 1. Ritkán
- □ 2. Néha
- □ 3. Gyakran
- □ 4. Szinte mindig

Hivatkozás: Kovács M, Makkos A, Weintraut R, Karádi K, Janszky J, Kovács N. Prevalence Of Anxiety Among Hungarian Subjects With Parkinson's Disease. Behav Neurol 2017;2017:1470149

The Parkinson Anxiety Scale (PAS); English version

A. Persistent anxiety
Please mark one circle for each item below

In the past four weeks, to what extent did you experience the following symptoms?

A.1. Feeling anxious or nervous
- o Not at all, or never
- o Very mild, or rarely
- o Mild, or sometimes
- o Moderate, or often
- o Severe, or (nearly) always

A.2. Feeling tense or stressed
- o Not at all, or never
- o Very mild, or rarely
- o Mild, or sometimes
- o Moderate, or often
- o Severe, or (nearly) always

A.3. Being unable to relax
- o Not at all, or never
- o Very mild, or rarely
- o Mild, or sometimes

34

- o Moderate, or often
- o Severe, or (nearly) always

A.4. Excessive worrying about everyday matters
- o Not at all, or never
- o Very mild, or rarely
- o Mild, or sometimes
- o Moderate, or often
- o Severe, or (nearly) always

A.5. Fear of something bad, or even the worst, happening
- o Not at all, or never
- o Very mild, or rarely
- o Mild, or sometimes
- o Moderate, or often
- o Severe, or (nearly) always

B. Episodic anxiety
Please mark one circle for each item below

In the past four weeks, did you experience episodes of the following symptoms?

B.1. Panic or intense fear
- o Never
- o Rarely
- o Sometimes
- o Often
- o Nearly always

B.2. Shortness of breath

o Never
o Rarely
o Sometimes
o Often
o Nearly always

B.3. Heart palpitations or heart beating fast (not related to physical effort or activity)

o Never
o Rarely
o Sometimes
o Often
o Nearly always

B.4. Fear of losing control

o Never
o Rarely
o Sometimes
o Often
o Nearly always

<u>C. Avoidance behavior</u>

Please mark one circle for each item below

In the past four weeks, to what extent did you fear or avoid the following situations?

C.1. Social situations (where one may be observed, or evaluated by others, such as speaking in public, or talking to unknown people)

o Never
o Rarely
o Sometimes
o Often
o Nearly always

C.2. Public settings (situations from which it may be difficult or embarrassing to escape, such as queues or lines, crowds, bridges, or public

transportation)
o Never
o Rarely
o Sometimes
o Often
o Nearly always
36

C.3. Specific objects or situations (such as flying, heights, spiders or other animals, needles, or blood)

o Never
o Rarely
o Sometimes
o Often
o Nearly always

Source: Leentjens AF, Dujardin K, Pontone GM, Starkstein SE, Weintraub D, Martinez-Martin P. The Parkinson Anxiety Scale (PAS): development and validation of a new anxiety scale. Mov Disord 2014;29(8):1035-1043.
